# Stability of Cocaine, Opiates, and Metabolites in Dried Saliva Spots

**DOI:** 10.3390/molecules27030641

**Published:** 2022-01-19

**Authors:** Ema Almeida, Sofia Soares, Joana Gonçalves, Tiago Rosado, Nicolás Fernández, Jesus M. Rodilla, Luís A. Passarinha, Mário Barroso, Eugenia Gallardo

**Affiliations:** 1 Centro de Investigação em Ciências da Saúde, Universidade da Beira Interior (CICS-UBI), Av. Infante D. Henrique, 6201-556 Covilhã, Portugal; ema.almeida97@gmail.com (E.A.); sofia_soares_26@hotmail.com (S.S.); joanadgoncalves13@gmail.com (J.G.); tiagorosadofful@hotmail.com (T.R.); 2 Laboratório de Fármaco-Toxicologia, Ubimedical, Universidade da Beira Interior, Estrada Municipal 506, 6200-284 Covilhã, Portugal; 3 C4-Cloud Computing Competence Centre, Universidade da Beira Interior, 6200-000 Covilhã, Portugal; 4 Laboratorio de Asesoramiento Toxicológico Analítico (CENATOXA), Cátedra de Toxicología y Química Legal, Facultad de Farmacia y Bioquímica, Universidad de Buenos Aires, Junín 956, 7mo piso, Ciudad Autónoma de Buenos Aires (CABA), Buenos Aires C1113AAD, Argentina; nfernandez@ffyb.uba.ar; 5 Departamento de Química, Universidade da Beira Interior, FibEnTech-Materiais Fibrosos e Tecnologias Ambientais, Rua Marquês d’Ávila e Bolama, 6200-001 Covilhã, Portugal; rodilla@ubi.pt; 6 UCIBIO-Applied Molecular Biosciences Unit, Departamento de Química, Faculdade de Ciências e Tecnologia, Universidade NOVA de Lisboa, 1099-085 Caparica, Portugal; 7 Associate Laboratory i4HB-Institute for Health and Bioeconomy, NOVA School of Science and Technology, Universidade NOVA, 2819-516 Caparica, Portugal; 8 Serviço de Química e Toxicologia Forenses, Instituto de Medicina Legal e Ciências Forenses-Delegação do Sul, 1169-201 Lisboa, Portugal

**Keywords:** cocaine, opiates, oral fluid, dried saliva spots, GC–MS/MS, stability

## Abstract

Drug abuse still represents a global problem, and it is associated with an increased risk of diseases, injuries, and deaths. Cocaine (COC) and opiates are the most abused drugs and account for a significant number of fatalities. Therefore, it is important to develop methods capable of effectively identifying and quantifying these substances. The present study aims to evaluate the long-term stability of COC, ecgonine methylester (EME), benzoylecgonine (BEG), cocaethylene (COET), norcocaine (NCOC), morphine (MOR), codeine (COD) and 6-monoacetylmorphine (6-MAM) in oral fluid samples. The analytes of interest were isolated from the matrix (50 µL) using the dried saliva spots (DSS) sampling approach and were subsequently analyzed by gas chromatography coupled with tandem mass spectrometry (GC–MS/MS). The parameters that could influence the stability of the target compounds were studied, and these were storage temperature, light, use of preservatives (and respective concentrations), and time. The effects of each parameter were evaluated using the design of experiments (DOE) approach. The stability of the target analytes was improved when the DSS were stored at room temperature, in the presence of light and using 1% sodium fluoride. The best conditions were then adopted for the DSS storage and long-term stability was assessed. COD was only stable for 1 day, EME was stable for 3 days, COC, COET, NCOC and 6-MAM were stable for 7 days, MOR for 14 days and BEG remained stable throughout the study (136 days). This is the first study that evaluates the stability of these compounds in oral fluid samples after application in DSS cards, and optimizes the conditions in order to improve their stability.

## 1. Introduction

Cocaine (benzoylmethylecgonine; COC) is the major alkaloid of *Erythroxylum coca* [1] and its first use dates back more than 4500 years ago, when coca leaves were used for religious and ritual ceremonies by the Andean civilizations [2]. Currently, COC is one of the most widely used stimulants and illicit drugs in Europe [2], and since its popularity has increased in recent years it is no longer considered an “elite drug” [3]. In 2018, COC and crack (a free base form of cocaine) were the second most used illicit drug group in Europe (10%), with an estimated number of 18 million consumers [4]. Its consumption is, however, associated with numerous health problems such as neurological impairment, cardiovascular disorders, as well as social problems and ultimately death [5,6,7]. 

Opiates, both licit and illicit, are another class of drugs commonly consumed worldwide and their use has been increasing in recent years [6]. According to the 2021 report of the European Monitoring Centre for Drugs and Drug Addiction (EMCDDA) opiates are responsible for about 76% of fatal overdoses [5]. Opiates use can also cause problems in the cardiovascular, nervous, respiratory, and gastrointestinal systems [8,9].

In recent years, the use of alternative specimens to blood and urine, to assess drug exposure become a trend in forensic toxicology [10,11,12]. One of those samples is oral fluid, presenting several advantages, namely the easy and non-invasive collection, reduced opportunity to tamper with, and since only the ‘free’ drug is present by passive diffusion from the blood circulation it efficiently reflects drug activity [11,13,14,15]. However, oral fluid has a small detection window when compared to other biological specimens [11,12,13,14,15]. Numerous methods have been published for the determination and identification of COC, opiates, and their metabolites in oral fluid, using different extraction techniques, such as the classical solid-phase extraction (SPE) [16,17,18,19,20,21], liquid–liquid extraction (LLE) [22,23], and protein precipitation (PP) [24,25,26,27] approaches, or more recently miniaturized techniques, for instance microextraction by packed sorbent (MEPS) [28,29]. Concerning chromatographic methods, the most commonly used is liquid chromatography (LC) coupled to tandem mass spectrometry (MS/MS) [13,17,18,19,20,23,24,25,26,30,31,32,33], but gas chromatography coupled to mass spectrometry (GC–MS) [13,16,22,32] and ultra-high-performance liquid chromatography (UHPLC) coupled to MS/MS have also been reported [13,21,27,28,32,34,35,36].

The dried matrix spots (DMS) sampling approach is acknowledged as a collection technique, simplifying transport and storage, since a liquid matrix is dried on a filter paper [37,38]. However, DMS is also used as an extraction technique involving the application of 50–100 µL of sample onto a filter paper and subsequently dried, after which a spot is cut and transferred to a tube containing an extracting solvent [38]. Usually the extraction occurs in a fast way under agitation [37,38]. Dried saliva spots (DSS), a subtype of DMS, have recently been proven as an excellent alternative to the classic techniques [39]. The use of DSS in bioanalysis was first reported for the determination and quantification of lidocaine by LC-MS/MS [40]. Since then, DSS has been successfully applied for the determination of D- and L-lactic acid in diabetic patients [41], antiepileptic drugs [42], methadone and its metabolite EDDP [43], and antipsychotics [44]. The DSS approach provides a low cost analysis, using reduced sample volumes, with low risk of contamination as well as great long-term stability of analytes [45].

The present work aims to evaluate the different conditions that may affect the stability of COC, ecgonine methylester (EME), benzoylecgonine (BEG), cocaethylene (COET), norcocaine (NCOC), morphine (MOR), codeine (COD), and 6-monoacetylmorphine (6-MAM) using the DSS sampling approach. Additionally, after the selection of the conditions that would improve stability, a study was performed in order to measure the time interval during which they were stable. 

To the best of our knowledge, this is the first study to determine the stability of these drugs of abuse in oral fluid samples using the DSS technique, and simultaneously evaluating the best conditions to maximize their stability.

## 2. Results and Discussion

### 2.1. Optimization of the Stability Protocol

In order to study the suitability of three different preservatives (ascorbic acid, sodium fluoride, and sodium azide) capable of improving COC, opiates, and their metabolites’ stability, the design of experiments (DOE) approach was used. This statistical tool can be used to optimize an experimental procedure and understand which are the optimal conditions [46,47,48]. This is possible because DOE not only allows a multivariate study of the different factors that can influence the response, but also evaluates the way these factors will interact with each other [46,47,48,49]. A two-level full factorial design was applied and four different factors were studied (2^4^), each of them at two levels: temperature (refrigerator (4 °C) or room), light (presence or absence), preservative concentration level [low (300 ng/mL, 1% and 0.1% for ascorbic acid, sodium fluoride, and sodium azide, respectively) or high (600 ng/mL, 2% and 0.2% for ascorbic acid, sodium fluoride, and sodium azide, respectively)] and storage time (1 or 7 days). The influence of light was studied using regular laboratory lamps, for dried spots placed either in the laboratory bench or in the refrigerator. To study the absence of light, the spots were protected by placing them inside of card boxes. Table 1 represents the experimental matrix followed. Data analysis was performed in MINITAB^®^, version 17, and the response was given by the relative peak area (compound absolute peak area/IS absolute peak area).

**Figure 1:**
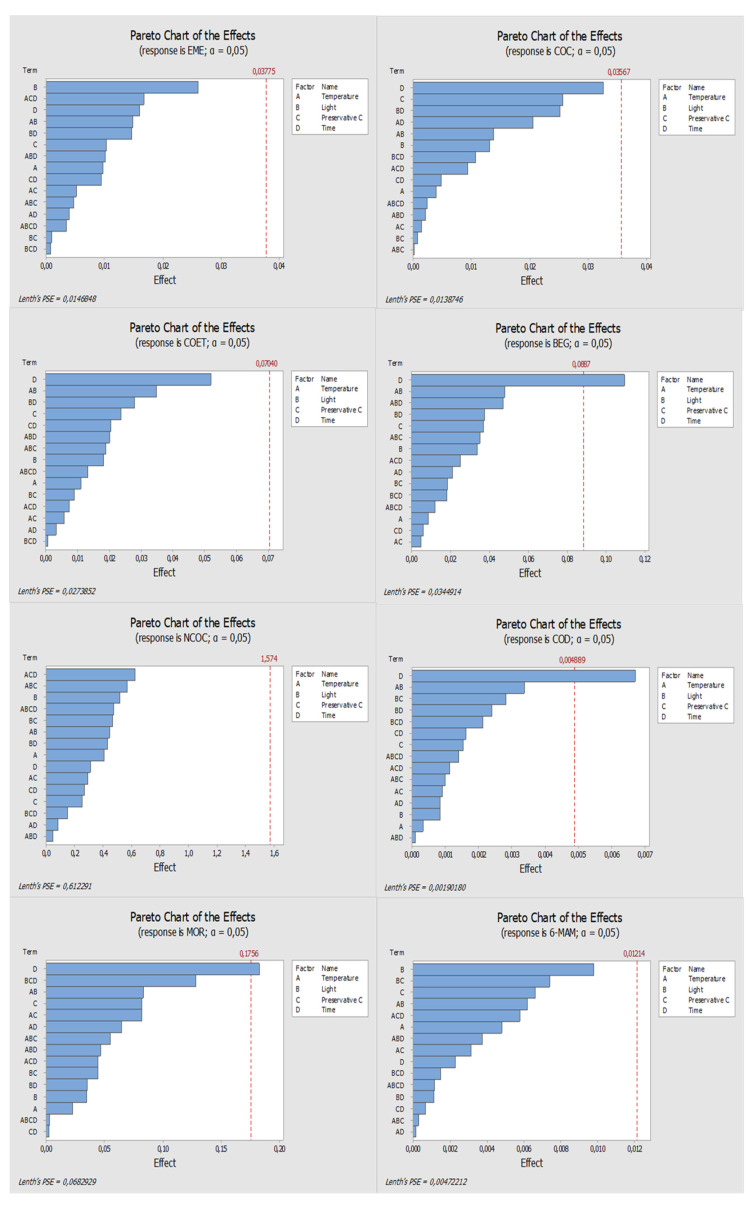
Pareto diagrams of the compounds under study, obtained when ascorbic acid was used as the preservative. The blue bars represent the effects of factors or a combination of factors: A—Temperature, B—Light, C—Preservative, and D—Time.

**Figure 2:**
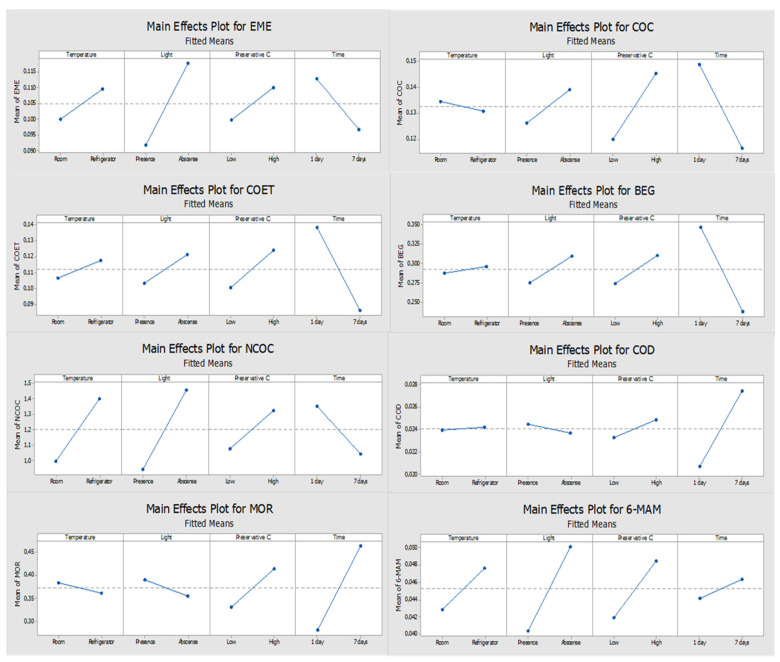
Main effects plots of the compounds when ascorbic acid was used as the preservative; The slope of the lines represents the effect of the response for the factors under study: temperature (room or refrigerator (4 °C)), light (presence or absence), preservative concentration level (low or high), and storage time (1 or 7 days).

**Figure 3:**
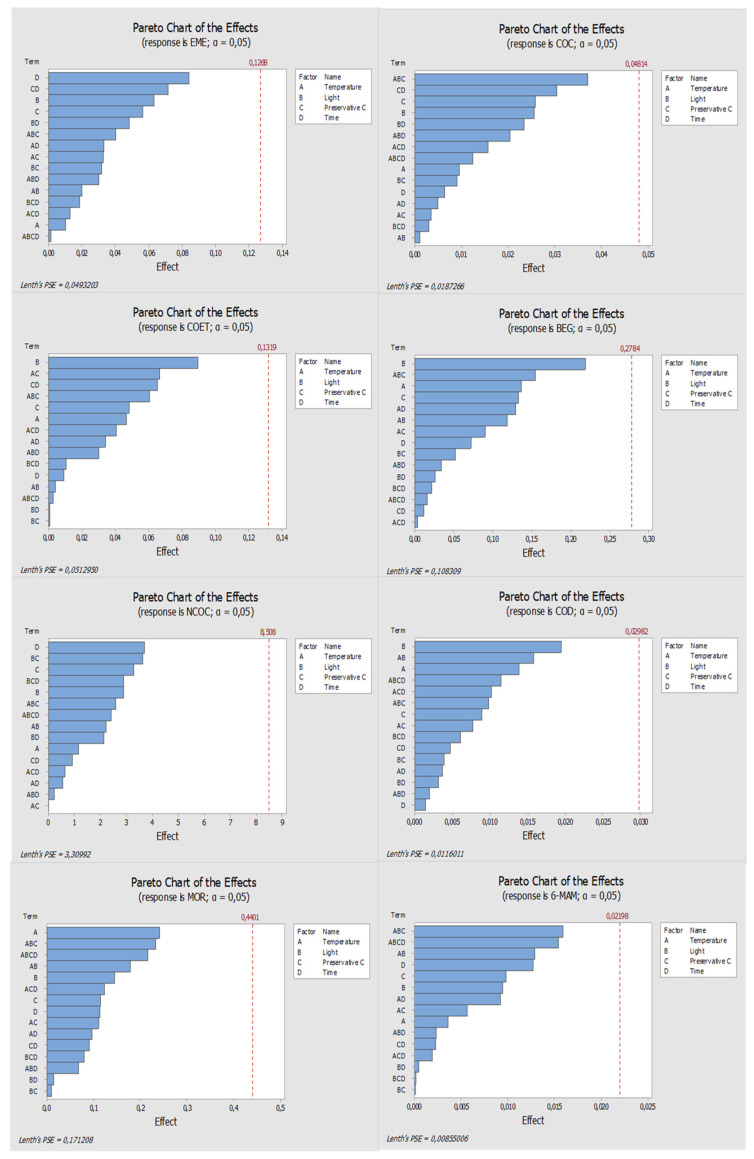
Pareto diagrams of the compounds under study, obtained when sodium fluoride was used as the preservative. The blue bars represent the effects of factors or a combination of factors: A—Temperature, B—Light, C—Preservative, and D—Time.

**Figure 4:**
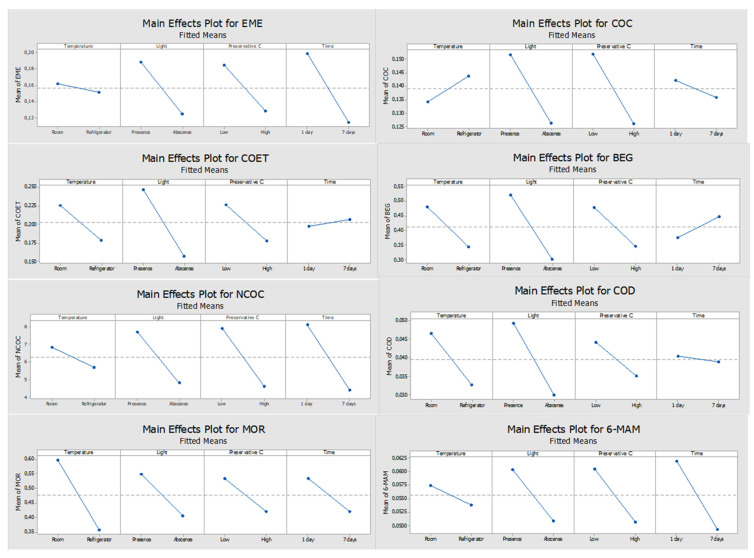
Main effects plots of the compounds when sodium fluoride was used as the preservative; The slope of the lines represents the effect of the response for the factors under study: temperature (room or refrigerator (4 °C)), light (presence or absence), preservative concentration level (low or high), and storage time (1 or 7 days).

Figure 1, Figure 2, Figure 3 and Figure 4 show the results obtained with the multivariate study matrix. According to Figure 1, one can observe that time is a parameter with a significant influence on the stability of BEG, COD, and MOR when ascorbic acid is used. For the remaining compounds, none of the evaluated factors revealed a significant influence, since none of them crosses the 5% significance line. By analyzing the main effects plots (Figure 2), it is possible to observe that most of the compounds present apparently better response when stored refrigerated, although not significant, except for COC and MOR. Most of the target analytes also present a slightly better response in the absence of light, with exception of COD and MOR. Additionally, all compounds present greater response when a high concentration (600 ng/mL) of ascorbic acid is used. Moreover, the compounds present a lower response after 7 days in the DSS with a significant decrease shown for BEG. The exceptions were observed for COD, MOR, and 6-MAM. Although time was not a significant parameter for 6-MAM, it had a significant influence for COD and MOR which presented better responses after 7 days.

Regarding sodium fluoride, it should be noted that none of the parameters under study had statistical significance on the compounds’ response, which means that the response is not affected by any of these factors (Figure 3). With this preservative, it is feasible to state that although without statistical significance, in general the best responses occurred when the conditions were room temperature, presence of light and low concentration (1%) of sodium fluoride (Figure 4). An exception was observed only for COC which presented better response under refrigeration conditions, but once again, no studied parameters revealed a significant influence on the results.

**Figure 5:**
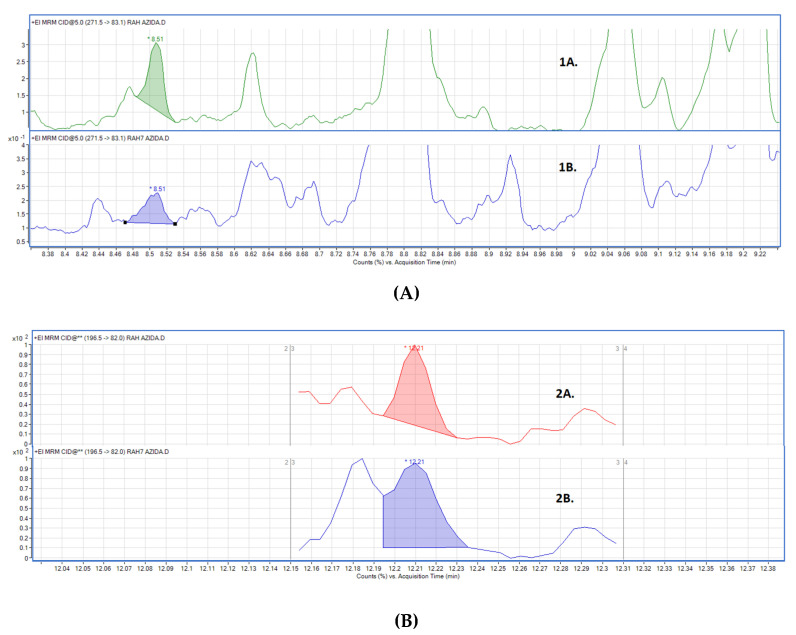
Chromatograms obtained for EME (**A**) and COET (**B**) for refrigerator temperature, absence of light, high concentration of preservative, day 1 (1A and 2A), and day 7 (1B and 2B) using sodium azide.

Lastly sodium azide was also studied as possible preservative, however its use resulted in a poor chromatographic resolution of the signals (Figure 5). For this reason, sodium azide was discarded and, therefore, DOE results were not considered.

Furthermore, and in order to determine the best preservative for the method, a comparison between the responses obtained for ascorbic acid and sodium fluoride was made, including all conditions under study. This allowed concluding that the best preservative was sodium fluoride, as it presented better responses to most conditions and compounds. This is also supported by the fact that no parameter, including time of storage, had a significant influence on the response. When ascorbic acid was adopted the situation was different, and time of storage revealed a significant influence on three compounds, with a significant loss observed for BEG after 7 days. It is possible to affirm that according to the multivariate study, when sodium fluoride is present, none of the other parameters, including preservative concentration, had a significant influence on the compounds’ responses, and no significant variations were observed after 7 days of storage. The latter is in accordance with the goal of the present work, which was the improvement of stability. 

Regarding the ideal conditions to adopt, and according to Pareto diagrams and by the slope of the main effects’ plots obtained when this agent was used, the best responses occurred when DSS were stored at room temperature for all the analytes except COC, in the presence of light, and using 1% of sodium fluoride. However, none of these parameters (temperature and light) had a significant influence on the response. Then, these settings were adopted for the long-term stability assay of the target analytes.

### 2.2. Assay without Preservative 

To study the stability of the compounds under the same conditions that were evaluated in the DOE, but without addition of any preservative, an assay was carried out. Thus, the oral fluid fortified with the compounds was applied to the spots, which were stored in the conditions under analysis. After 1 and 7 days, the extraction process was carried out as referred to in the topic of sample preparation, and the response was calculated for each of the compounds for all conditions. To compare the results obtained for each of the conditions, the average was calculated between the responses obtained for day 1 and day 7. These results are shown in Table 2.

Analyzing Table 2, it is possible to verify that for EME, COC, BEG, COD, and 6-MAM there are no differences between the average values obtained with the different conditions, which indicates that for these analytes, temperature, and light have no influence in their stability. In the case of COET, the best condition is number 4 (room temperature and absence of light). For NCOC, better results were obtained at room temperature, either with or without light, and the best response for MOR occurred when the test was performed in the absence of light, not depending on the storage temperature. Thus, for the assay without preservative it was determined that the best conditions were room temperature and absence of light.

Comparing the responses obtained for each analyte with the addition of 1% sodium fluoride at room temperature in the presence of light (the best conditions with preservative), with the responses achieved without preservative at room temperatures in the absence of light (the best conditions without preservative) (Table 3), it is possible to observe that when sodium fluoride was added the responses were higher for BEG, EME, COC, and MOR after 1 and 7 days of storage. Most compounds presented greater stability when sodium fluoride was added, and as such its addition to the samples is thus justified. 

### 2.3. Long-Term Stability 

Long-term stability was assessed over a period of 136 days, and the samples were extracted and analyzed after 1, 3, 7, 14, 21, 28, 44, 112, 121, and 136 days of storage under the optimized conditions (presence of light and room temperature). For each of these days, a mixture of oral fluid with the compounds under study at 100 ng/mL and sodium fluoride (1%), was prepared and applied to spots in triplicate (*n* = 3). For each spot, the chromatograms obtained were analyzed and the areas of the compound and the respective IS were extracted to calculate the response (relative peak area) for every compound in all spots. The coefficients of variation (CV) obtained were lower than 20% for all days and compounds. To assess the losses of each analyte throughout the study, the variation compared to a fresh sample (day 1) was calculated as a percentage. Table 4 presents these data.

**Figure 6:**
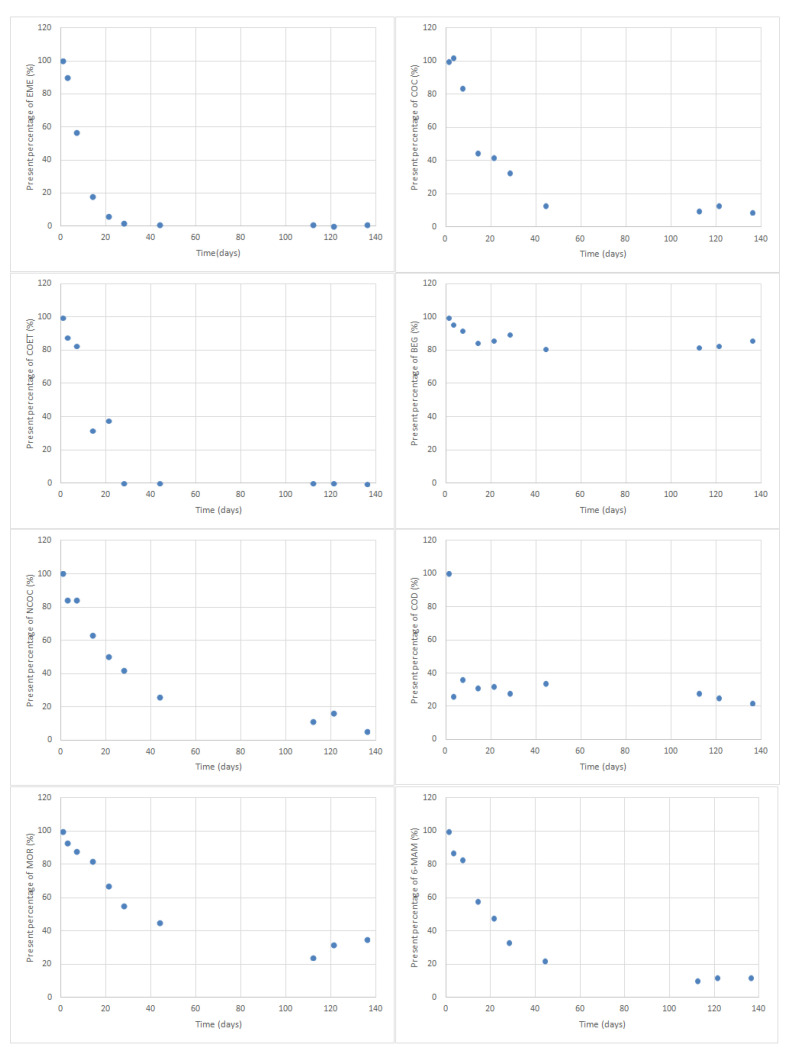
Long-term stability of analytes under study. The graphs represent the percentage of the compounds in the DSS samples as a function of time.

The percentage of analyte that was present in the extracts on each day of the study was determined for the purpose of assessing long-term stability and was calculated based on the verified variations from day 1, assuming that on this day the value was 100% for all analytes. The obtained results are shown in Figure 6.

A variation higher than 20% of the initial drug response was considered as an indicator of possible instability; therefore, to affirm that a compound is stable, the percentage should be between 80 and 120%. Thus, analyzing the graphics in Figure 6, it is possible to observe the stability of each analyte under the optimized conditions. Thus, EME was stable for 3 days, COC, COET, and NCOC remained stable for 7 days and BEG was shown to be stable throughout the study. Even if only BEG has a stability greater than 1 week, this method can be very useful since although COC and other metabolites disappear, BEG remains stable for at least 136 days, which allows the detection of COC consumption, because this metabolite can only be found in the oral fluid after consumption. Regarding opiates, COD was the compound that showed lower stability, just 1 day, followed by 6-MAM that remained stable for 7 days and MOR whose stability was 14 days.

The present work is the first to determine their stability in DSS. This approach presents several advantages relatively to other approaches, namely in what concerns storing conditions. Indeed, storing DSS cards at room temperature is easier than storing neat samples under refrigeration or lower temperatures. In addition, extracting the analytes from the DSS is in general easier than extracting neat or buffered oral fluid samples. 

Several stability studies exist in the scientific literature under different storage conditions, using different collection devices, preservatives and/or stabilizers. In these studies the stability of these compounds has been evaluated in neat or buffered oral fluid [21,22,36,50,51,52,53,54]. For instance, Ventura et al. [50] evaluated the short- and long-term stability of opiates and COC in oral fluid specimens under different storage conditions (−20, 4, 25 and 37 °C) with and without the addition of a citrate buffering solution. The authors concluded that BEG, COD and MOR were stable for 7 days at 25 and 37 °C and for at least 2 months at 4 and −20 °C, and also that COC and 6-MAM were stable for less than 7 days at 25 and 37 °C when oral fluid was not buffered. They also concluded that the addition of the citrate buffer increased the stability of COC and 6-MAM, preventing degradation for at least 7 days when stored at 25 and 37 °C and up to 2 months at 4 and −20 °C. These results are generally in agreement with those herein presented, demonstrating that the addition of a preservative, in this case citrate buffer, prevents the degradation of some compounds, increasing their stability.

Langel et al. [51] studied the stability of several drugs of abuse including COC, MOR, and COD in oral fluid samples after 0, 14, and 28 days of storage at −18 °C, using different buffered oral fluid collection devices. On most devices, although with some differences, the analytes remained stable for the 28 days of the study. 

Similarly, Lund et al. [52] tested the stability of different drugs of abuse in oral fluid samples using Intercept^®^ and StatSture Saliva Sampler™, after 1 week of storage at 20 and 4 °C and after 3 and 11 months at −20 °C. Opiates, with the exception of 6-MAM, were stable for the first week at all tested temperatures, which is in accordance with the results obtained in the present study for MOR. With the herein described DSS method 6-MAM was stable for a week but COD was stable for one day only. The authors also observed that the concentration of COC decreases over the first week, being this decrease more accentuated at room temperature. In contrast, BEG concentration increases during the same period of time probably due to COC degradation. These results agree with those presented in this manuscript. In the same way, Cohier et al. [53] studied the stability of opiates, COC, and metabolites in oral fluid samples, collected with Quantisal^®^ and Certus^®^ and stored under different temperatures (room temperature, −20 and 4 °C) for 14 days. At −20 °C, the results obtained for stability were less than 7 days for COD and 7 days for EME in Quantisal^®^, 14 days for BEG in Certus^®^ and 14 days for COC, MOR, and 6-MAM on both collectors. Regarding the assay at 4 °C, with the exception of COD, the compounds remained stable for 14 days in both devices. Finally, at room temperature, COC and COD showed stability of less than 7 days, 6-MAM, EME, and BEG remained stable for 7 days and MOR for 14 days. The results obtained for opiates are in line with those obtained with sodium fluoride in the present study; however, for COC and BEG, higher stability was achieved at room temperature.

Valen et al. [36] quantified 21 drugs in oral fluid including COC, COD, MOR, and 6-MAM and evaluated their stability for 30 days at −20, 4, and 18 °C after collection with Intercept^®^ and Quantisal^®^ devices. At −20 °C all analytes remained stable after 30 days of storage for both devices. At 4 °C, opiates were stable during the 30 days of the study, while the COC showed stability of 30 days for Quantisal^®^ and 7 days for Intercept^®^. Regarding the study of stability at room temperature, the results obtained for Intercept^®^ were 1, 7, and 30 days for 6-MAM, MOR, and COD, and less than 1 day for COC. Comparing these results with the described method, it is possible to verify that using this device the stability of COD is increased, but for the other three analytes, the DSS approach originated higher stability. With Quantisal^®^, the authors reported 30 days stability for 6-MAM and COD, 7 days for MOR, and 1 day for COC. Comparing the results obtained at room temperature for Quantisal^®^ with the present work, it is possible to verify that the buffer present in this collection device increases the stability of COD and 6-MAM, but for COC and MOR the stability is greater with the sodium fluoride DSS technique described in this work.

Fabresse et al. [21], developed a method for the detection of 10 illicit drugs in oral fluid samples collected with the FLOQSwabs™ which does not contain an elution buffer. The stability of the compounds was evaluated over 7 days of storage at 4 °C. MOR and COC remained stable for 3 days, BEG for 7 days, while COD and 6-MAM degraded before 3 days of storage, proving to be the most unstable compounds. Analyzing these results, it is possible to conclude that the stability of the compounds, with the exception of COD, is higher when the oral fluid is stored in DSS at room temperature and with the addition of sodium fluoride, than when it is collected and stored in the FLOQSwabs™ at 4 °C.

Lastly, Marchei, et al. [54] investigated the stability of commonly used drugs of abuse and their main metabolites in oral fluid with, and without the presence of an alternative stabilizing buffer (M3 Reagent Buffer^®^), for 1 year of storage at three different temperatures (room temperature, −20, and 4 °C). In M3 samples, the authors concluded that all compounds remained stable for 1 year at −20 °C and that 6-MAM was stable in neat oral fluid at −20 °C for 30 days, up to 14 days at 4 °C, but it degraded in less than 1 day at room temperature. With the M3 buffer, 6-MAM showed no degradation until 90 days at 4 °C and was stable for the first 7 days at room temperature. They also concluded that MOR is stable for 1 day at room temperature, 14 days at 4 °C, and 30 days when stored at −20 °C. In M3 samples, MOR stability increased to 14 and 90 days at room temperature and 4 °C, respectively. COD is stable in neat oral fluid samples up to 30 days at room temperature, up to 180 days at 4 °C and 1 year at −20 °C. In the assay with M3 buffer COD was stable for 1 year at all tested temperatures. COC concentration in neat oral fluid decreased around 26% after the first day at room temperature and started to decline after 7 days at 4 °C and after 90 days at −20 °C. The stability of BEG at room temperature, as verified for COC, was less than 1 day and when stored at 4 °C remained stable for 14 days, and at −20 °C for 1 year. Both COC and BEG, were stable for 1 year at all temperatures when M3 buffer was added to the oral fluid samples. Comparing the stability at room temperature achieved with buffer M3, with the stability achieved with sodium fluoride in the present assay for MOR and 6-MAM, the results were similar (14 and 7 days, respectively), however for COD, BEG, and COC higher stability was obtained with the M3 buffer.

## 3. Materials and Methods

### 3.1. Reagents and Standards

The analytical standards of COC, EME, BEG, NCOC, and COET were obtained from Sigma-Aldrich (St. Louis, MO, USA). The internal standards (IS) of COC-d3, EME-d3, and BEG-d3, the analytical standards of COD, MOR, and 6-MAM as well as the IS of COD-d3 and 6-MAM-d3, were purchased from Sigma-Aldrich (Lisbon, Portugal). All of these stock solutions were obtained at a concentration of 1 mg/mL, with the exception of EME-d3 which was obtained at 100 µg/mL. For the compounds that did not have a respective deuterated IS (NCOC and COET), COC-d3 was used.

Ascorbic acid was acquired from Fisher Scientific (Loughborough, UK), sodium fluoride from Sigma-Aldrich (Sintra, Portugal), and sodium azide from Panreac Quimica (Barcelona, Spain). Deionized water was obtained from a Milli-Q System (Millipore, Billerica, MA, USA). N-methyl-N-(trimethylsilyl) trifluoroacetamide (MSTFA) and trimethylchlorosilane (TMCS) were acquired from Macherey-Nagel (Düren, Germany). Whatman™ 903 protein save cards were purchased from Sigma-Aldrich (Sintra, Portugal).

Working solutions for COC and metabolites were prepared by diluting the stock solutions with methanol (Merck Co., Darmstadt, Germany) to the final concentration of 500 ng/mL for COC, BEG, EME, COET, and NCOC and 1 µg/mL for ISs. Regarding the working solutions of opiates, the dilution of the stock solutions was performed with acetonitrile (Carlo Erba Reagents, Val-de-Reuil, France) to a final concentration of 2.5 µg/mL for MOR, COD, and 6-MAM and 500 ng/mL for ISs. All work and stock solutions were stored in the absence of light at 4 °C.

### 3.2. Biological Specimens

Blank oral fluid samples used in the present work were supplied by laboratory staff. All oral fluid samples were collected by spitting, without the use of any specific collection device.

### 3.3. Gas Chromatographic and Mass Spectrometric Conditions

An HP 7890A gas chromatography system (GC) equipped with 7000B triple quadrupole mass spectrometer (MS/MS), both from Agilent Technologies (Waldbronn, Germany), coupled to a MPS2 autosampler and a PTV-injector from Gerstel (Mülheim an der Ruhr, Germany) was used for the chromatographic analysis. The analytes’ separation was achieved with a capillary column (30 m × 0.25 mm I.D., 0.25-µm film thickness) with 5% phenylmethylsiloxane (HP-5 MS), supplied by J&W Scientific (Folsom, CA, USA).

The initial oven temperature was held at 90 °C for 2 min, then increased to 300 °C at the rate of 20 °C/min and held for 3 min, giving a total run time of 15.50 min. The injector and transfer line temperature were set at 220 and 280 °C, respectively. The sample was injected in splitless mode and the flow of helium (carrier gas) was held constant at 0.8 mL/min. The mass spectrometer operated with a filament current of 35 μA and electron energy of 70 eV in the positive electron ionization mode, and the flow rate of the collision gas (nitrogen) was set at 2.5 mL/min. 

Data was acquired in the multiple reaction monitoring (MRM) mode using the MassHunter WorkStation Acquisition Software rev. B.02.01 (Agilent Technologies). Optimization of the tandem mass spectrometry conditions, was performed by injection of the derivatized standard solution at different collision energy and dwell times. The transitions were chosen based on selectivity, signals abundance and signal-to-noise ratio in matrix extracts. Table 5 shows the retention times, quantifier and qualifier transitions, collision energies, and dwell times selected for each compound.

Data on method validation is provided in Supplementary Material.

### 3.4. Sample Preparation

The procedure for the extraction of COC, opiates, and its metabolites was as follows: after homogenization, 50 µL of oral fluid with preservative were applied in the Whatman™ 903 protein saver card and left to dry for 12 h. Subsequently, the spots of each sample were cut and placed in a tube to which 3 mL of methanol, 10 µL of the IS solution at 1 µg/mL (COC-d3, EME-d3, BEG-d3) and 20 µL of the IS solution at 500 ng/mL (COD-d3, 6-MAM-d3) were added. The tubes were placed on a roller mixer for 5 min at room temperature and then centrifuged for 15 min at 3500 rpm. The extract was submitted to a gentle stream of nitrogen until dryness and then it was derivatized with 50 µL of MSTFA with 5% of TMCS for 2 min in a microwave oven at 800 W [55]. Finally, a 3-µL aliquot (splitless mode) was injected into the GC–MS/MS system.

## 4. Conclusions

The present work allowed for the establishment of the best conditions to improve the stability of COC, opiates, and metabolites in oral fluid samples, when applied in Dried Saliva Spots (DSS). The parameters that could influence the stability of the compounds were evaluated and optimized with the help of the Design of Experiments (DOE) approach. This statistical tool proved to be an excellent instrument in the optimization of storage conditions, allowing to reduce the number of experiments.

Thus, through the analysis of the results obtained by DOE, it was possible to conclude that of the preservatives under study, sodium fluoride at 1% was the one that appeared to improve the stability of the analytes in oral fluid samples stored in the DSS. Additionally, the best conditions of storage were room temperature and presence of light (regular laboratory lamps). 

In addition, by comparing the results obtained with sodium fluoride, with those of the study without preservative, it was possible to verify the importance of its addition, since it increases the responses of the analytes and consequently its stability after 7 days of storage.

For the long-term stability, the analytes proved to be stable at room temperature and in presence of light for 7 days except for COD, EME, MOR, and BEG, whose stability was 1, 3, 14, and 136 days, respectively.

This is the first time that DSS technique combined with GS–MS/MS is used to detect these drugs of abuse in oral fluid samples and to evaluate their stability in this biological specimen.

## Figures and Tables

**Table 1:** Experimental matrix.

Run Order	Temperature	Light	Preservative	Time
1	Refrigerator	Presence	Low	1 Day
2	Refrigerator	Presence	Low	7 Days
3	Refrigerator	Presence	High	7 Days
4	Refrigerator	Absence	Low	1 Day
5	Room	Presence	High	7 Days
6	Room	Absence	Low	7 Days
7	Room	Absence	High	7 Days
8	Room	Absence	Low	1 Day
9	Refrigerator	Absence	High	7 Days
10	Room	Absence	High	1 Day
11	Room	Presence	Low	7 Days
12	Refrigerator	Absence	Low	7 Days
13	Room	Presence	High	1 Day
14	Refrigerator	Presence	High	1 Day
15	Room	Presence	Low	1 Day
16	Refrigerator	Absence	High	1 Day

**Table 2:** Responses obtained for the assay without preservative.

Condition	EME	COC	COET	BEG	NCOC	COD	MOR	6-MAM
1	0.16 ± 0.008	0.17 ± 0.002	0.14 ± 0.019	0.36 ± 0.050	0.03 ± 0.008	0.02 ± 0.004	0.21 ± 0.015	0.03 ± 0.006
2	0.16 ± 0.011	0.18 ± 0.021	0.24 ± 0.010	0.30 ± 0.005	0.06 ± 0.007	0.03 ± 0.021	0.39 ± 0.099	0.03 ± 0.008
3	0.15 ± 0.034	0.18 ± 0.002	0.28 ± 0.034	0.36 ± 0.04	0.07 ± 0.005	0.02 ± 0.0006	0.21 ± 0.009	0.03 ± 0.005
4	0.18 ± 0.031	0.17 ± 0.058	0.29 ± 0.016	0.28 ± 0.071	0.08 ± 0.002	0.02 ± 0.009	0.46 ± 0.097	0.03 ± 0.0002

Mean values ± standard deviation. 1: refrigerator temperature, presence of light; 2: refrigerator temperature, absence of light; 3: room temperature, presence of light; and 4: room temperature, absence of light.

**Table 3:** Response of analytes with and without preservative after 1 and 7 days of storage under ideal conditions.

	Day	EME	COC	COET	BEG	NCOC	COD	MOR	6-MAM
Without preservative	1	0.16	0.23	0.29	0.31	0.09	0.02	0.35	0.03
	7	0.22	0.14	0.24	0.24	0.05	0.02	0.47	0.03
With preservative	1	0.54	0.35	0.34	1.23	0.13	0.04	0.88	0.04
	7	0.32	0.28	0.27	1.14	0.10	0.01	0.77	0.03

**Table 4:** Long-term stability (*n* = 3).

Analyte	Day	Concentration (ng/mL) *	CV (%)	Variation from Day 1 (%)
EME	1	105.38 ± 0.028	5.10	
	3	94.73 ± 0.084	17.1	−10.1
	7	60.52 ± 0.013	4.3	−42.6
	14	18.45 ± 0.009	9.5	−82.5
	21	6.67 ± 0.008	20.0	−93.7
	28	2.22 ± 0.001	10.5	−97.9
	44	1.20 ± 0.0007	10.7	−98.9
	112	0.91 ± 0.0006	12.3	−99.1
	121	0.29 ± 0.0001	9.6	−99.7
	136	0.58 ± 0.0002	8.1	−99.4
COC	1	86.28 ± 0.056	15.8	
	3	88.25 ± 0.054	15.1	2.3
	7	72.27 ± 0.035	11.9	−16.2
	14	38.90 ± 0.032	20.5	−54.9
	21	36.01 ± 0.024	16.6	−58.3
	28	28.48 ± 0.013	11.6	−67.0
	44	10.93 ± 0.005	10.4	−87.3
	112	8.72 ± 0.002	4.8	−89.9
	121	11.00 ± 0.007	15.1	−87.3
	136	7.67 ± 0.004	14.0	−91.2
COET	1	86.05 ± 0.053	16.3	
	3	75.45 ± 0.035	12.4	−12.3
	7	71.67 ± 0.042	15.3	−16.7
	14	27.82 ± 0.022	20.5	−67.7
	21	32.42 ± 0.022	17.9	−62.3
	28	30.96 ± 0.011	9.70	−64.0
	44	14.21 ± 0.010	17.8	−83.5
	112	7.76 ± 0.004	13.7	−91.0
	121	7.95 ± 0.006	19.0	−90.8
	136	2.15 ± 0.0008	10.2	−97.5
BEG	1	95.83 ± 0.230	18.9	
	3	92.24 ± 0.070	6.0	−3.7
	7	88.55 ± 0.183	16.3	−7.6
	14	81.23 ± 0.124	12.1	−15.2
	21	82.41 ± 0.135	13.0	−14.0
	28	85.95± 0.174	15.9	−10.3
	44	77.30 ± 0.034	3.5	−19.3
	112	78.40 ± 0.072	7.2	−18.2
	121	79.06 ± 0.055	5.4	−17.5
	136	82.85 ± 0.145	13.8	−13.5
NCOC	1	117.04 ± 0.018	14.8	
	3	98.46 ± 0.019	18.1	−15.9
	7	98.44 ± 0.013	12.4	−15.9
	14	73.27 ± 0.014	18.6	−37.4
	21	58.82 ± 0.010	16.8	−49.7
	28	49.66 ± 0.004	7.9	−57.6
	44	29.92 ± 0.003	8.8	−74.4
	112	13.15 ± 0.002	16.2	−88.8
	121	18.33 ± 0.003	15.2	−84.3
	136	6.16 ± 0.0005	8.4	−94.7
COD	1	83.53 ± 0.006	15.8	
	3	22.54 ± 0.001	13.1	−73.9
	7	30.79 ± 0.001	11.0	−64.4
	14	26.52± 0.002	13.8	−69.3
	21	27.94 ± 0.002	18.1	−67.7
	28	27.85 ± 0.002	18.4	−67.8
	44	29.01 ± 0.002	12.3	−66.5
	112	24.14 ± 0.0002	2.0	−72.1
	121	21.67 ± 0.0004	4.3	−75.0
	136	18.86 ± 0.001	15.1	−78.2
MOR	1	89.11 ± 0.106	12.2	
	3	82.63 ± 0.010	12.4	−7.3
	7	78.45 ± 0.092	12.1	−12.0
	14	73.28 ± 0.024	3.4	−17.8
	21	59.83 ± 0.047	8.1	−32.9
	28	49.25 ± 0.035	7.4	−44.7
	44	39.75 ± 0.044	11.4	−55.4
	112	21.63 ± 0.032	15.2	−75.7
	121	28.19 ± 0.025	8.9	−68.4
	136	31.34 ± 0.052	17.0	−64.8
6-MAM	1	108.96 ± 0.003	8.0	
	3	94.91 ± 0.005	17.5	−12.9
	7	90.84 ± 0.005	16.7	−16.6
	14	62.66 ± 0.002	9.8	−42.5
	21	52.75 ± 0.003	19.5	−51.6
	28	35.48 ± 0.002	16.1	−67.4
	44	24.01 ± 0.0005	6.8	−78.0
	112	10.64 ± 0.0001	3.3	−90.2
	121	13.38 ± 0.0007	16.2	−87.7
	136	13.19 ± 0.0007	16.0	−87.9

CV: coefficient of variation; * mean values ± standard deviation.

**Table 5:** Retention times and selected transitions for the identification of analytes.

Analyte	Retention Time (min)	Transitions (m/z)	Collision Energy (eV)	Dwell Time (µs)
EME	8.51	271.5–83.1 * 181.3–82.0	5 10	50
EME-d3	8.50	274.5–86.1	10	50
COC	11.97	182.5–82.2 * 182.5–150.1	10 5	50
COC-d3	11.96	184.1–85.0	10	50
COET	12.22	196.5–82.0 * 196.5–150.2	10 5	50
BEG	12.25	239.6–82.2 * 239.6–122.2	15 15	50
BEG-d3	12.21	241.9–85.1	15	50
NCOC	12.33	178.1–105.1 * 178.1–135.1	15 10	50
COD	13.08	369.8–229.2 369.8–354.3 *	20 20	50
COD-d3	13.08	374.0–374.0	5	50
MOR	13.31	235.4–146.1 * 235.4–220.2	10 5	50
6-MAM	13.66	397.9–287.4 * 397.9–340.5	20 20	50
6-MAM-d3	13.65	402.4–402.4	5	50

* quantifier transition.

## Data Availability

Data is contained within the article.

## Supplementary Materials

The following are available online, Figure S1: Selectivity; Table S1: Linearity data; Table S2: Intraday precision and accuracy.
