# Supplementary material for: Stability of Cocaine, Opiates, and Metabolites in Dried Saliva Spots
Source: Molecules. 2022 Jan 19;27(3):641. doi: 10.3390/molecules27030641 (PMC8839019; doi:10.3390/molecules27030641)
Supplement: Supplementary file 1 [file molecules-27-00641-s001.zip › molecules-1518995-supplementary.pdf]

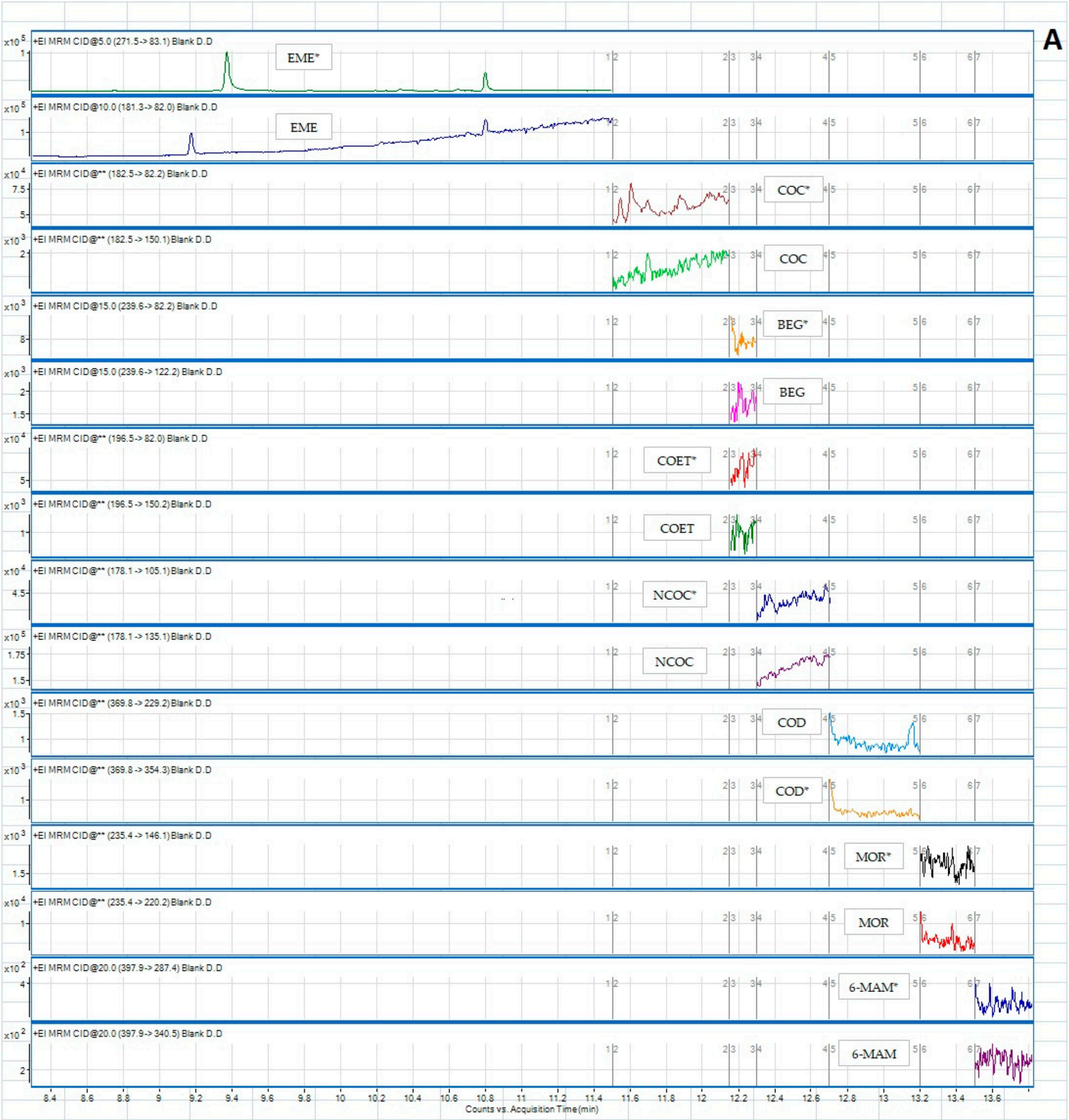
A
+EI MRM CID@5.0 (271.5 -> 83.1) Blank D.D
EME*
+EI MRM CID@10.0 (181.3 -> 82.0) Blank D.D
EME
+EI MRM CID@** (182.5 -> 82.2) Blank D.D
COC*
+EI MRM CID@** (182.5 -> 150.1) Blank D.D
COC
+EI MRM CID@15.0 (239.6 -> 82.2) Blank D.D
BEG*
+EI MRM CID@15.0 (239.6 -> 122.2) Blank D.D
BEG
+EI MRM CID@** (196.5 -> 82.0) Blank D.D
COET*
+EI MRM CID@** (196.5 -> 150.2) Blank D.D
COET
+EI MRM CID@** (178.1 -> 105.1) Blank D.D
NCOC*
+EI MRM CID@** (178.1 -> 135.1) Blank D.D
NCOC
+EI MRM CID@** (369.8 -> 229.2) Blank D.D
COD
+EI MRM CID@** (369.8 -> 354.3) Blank D.D
COD*
+EI MRM CID@** (235.4 -> 146.1) Blank D.D
MOR*
+EI MRM CID@** (235.4 -> 220.2) Blank D.D
MOR
+EI MRM CID@20.0 (397.9 -> 287.4) Blank D.D
6-MAM*
+EI MRM CID@20.0 (397.9 -> 340.5) Blank D.D
6-MAM
Counts vs. Acquisition Time (min)

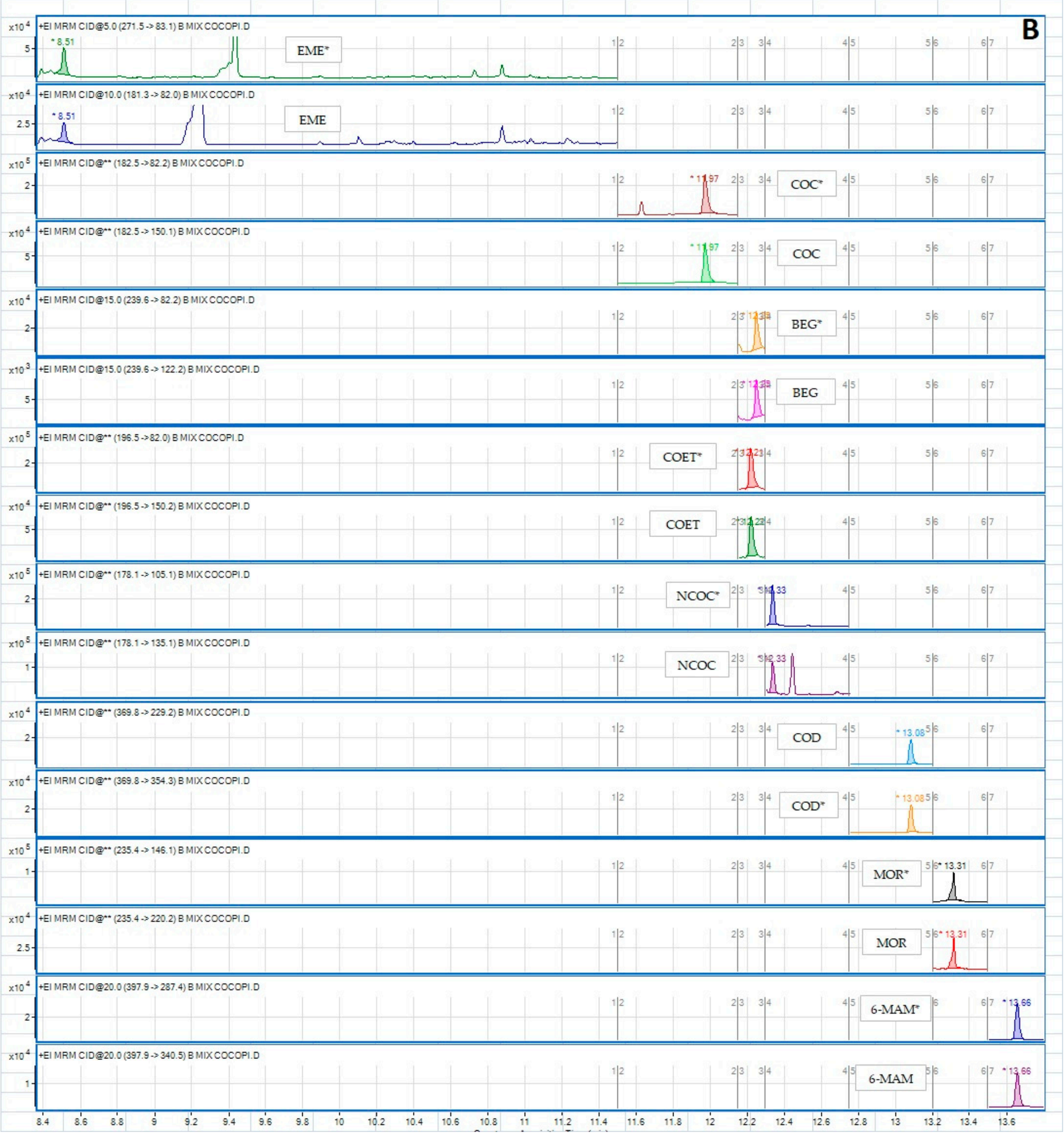

Figure S1. Selectivity (A—Blank sample; B—Spiked sample at 100 ng/mL)

Table S1. Linearity data

Analytes	Weight	Linear range (ng/mL)	Linearity		R^{2a}	LLOQ (ng/mL)
			Slope[a]	Intercept[a]		
**EME**	1/x	15-800	0.0012 ± 0.0009	-0.0044 ± 0.0012	0.9946 ± 0.0012	15
**COC**	1/x	10-800	0.0129 ± 0.0073	0.0744 ± 0.0587	0.9919 ± 0.0056	10
**COET**	1/x	20-800	0.0137 ± 0.0025	0.0779 ± 0.0438	0.9938 ± 0.0048	20
**BEG**	1/x	10-800	0.0169 ± 0.0007	-0.0390 ± 0.0112	0.9980 ± 0.0094	10
**NCOC**	1/x	15-800	0.0069 ± 0.0004	0.0063 ± 0.0054	0.9952 ± 0.0032	15
**COD**	1/x	20-300	0.0003 ± 0.0000	0.0071 ± 0.0042	0.9904 ± 0.0017	20
**MOR**	1/x	15-300	0.0021 ± 0.0006	-0.0115 ± 0.0094	0.9990 ± 0.0011	15
**6-MAM**	1/x	10-300	0.0003 ± 0.0000	-0.0018 ± 0.0013	0.9968 ± 0.0026	10

[a]Mean values ± standard deviation; **Quality controls:** 25, 40, 300 and 700 ng/mL (for COC and metabolites); 25, 40, 150 and 250 ng/mL (for opiates).

Table S2. Intraday precision and accuracy

Analytes	Spiked (ng/mL)	Intra-day (n = 6)	
		CV (%)	RE[a] (%)
**EME**	25	0.19	14.68
	40	0.53	14.88
	100	0.24	14.67
	300	0.88	14.60
	700	0.04	14.96
**COC**	25	14.76	-1.90
	40	4.41	1.86
	100	11.97	-10.75
	300	12.54	3.30
	700	0.49	14.09
**COET**	25	6.31	10.58
	40	6.10	9.24
	100	5.82	4.33
	300	3.59	10.68
	700	0.31	14.93
**BEG**	25	14.90	1.81
	40	4.36	-4.49
	100	1.05	-14.59
	300	3.61	-12.54
	700	9.08	-6.41
**NCOC**	25	3.13	14.63
	40	6.89	6.79
	100	11.99	-5.08
	300	12.74	12.19
	700	5.50	-12.42
**COD**	25	1.45	-11.98
	40	14.65	-9.39
	100	0.53	14.88
	150	4.69	11.68
	250	13.71	14.79
**MOR**	25	8.91	-8.91
	40	4.11	3.30
	100	3.94	10.73
	150	9.89	-0.08
	250	10.56	-2.34
**6-MAM**	25	2.23	12.27
	40	6.48	7.20
	100	0.20	14.70
	150	7.49	-3.51
	250	3.69	10.25

CV - coefficient of variation, RE - relative error; relative error [(measured concentration - spiked concentration/spiked concentration) × 100]
